# Evaluating the immunologically “cold” tumor microenvironment after treatment with immune checkpoint inhibitors utilizing PET imaging of CD4 + and CD8 + T cells in breast cancer mouse models

**DOI:** 10.1186/s13058-024-01844-3

**Published:** 2024-06-25

**Authors:** Yun Lu, Hailey A. Houson, Carlos A. Gallegos, Alessandro Mascioni, Fang Jia, Argin Aivazian, Patrick N. Song, Shannon E. Lynch, Tiara S. Napier, Ameer Mansur, Benjamin M. Larimer, Suzanne E. Lapi, Ariella B. Hanker, Anna G. Sorace

**Affiliations:** 1 Department of Radiology, University of Alabama at Birmingham, Birmingham, AL 35233 USA; 2 Graduate Biomedical Sciences, University of Alabama at Birmingham, Birmingham, AL 35233 USA; 3 Department of Biomedical Engineering, University of Alabama at Birmingham, Birmingham, AL 35233 USA; 4 O’Neal Comprehensive Cancer Center, University of Alabama at Birmingham, Birmingham, AL 35233 USA; 5 ImaginAb, Inc, Inglewood, CA 90301 USA; 6 Department of Chemistry, University of Alabama at Birmingham, Birmingham, AL 35233 USA; 7 Harold C. Simmons Comprehensive Cancer Center, UT Southwestern Medical Center, Dallas, TX 75390 USA; 8 Departments of Radiology and Biomedical Engineering, O’Neal Comprehensive Cancer Center, University of Alabama at Birmingham, Small Animal Imaging Facility, 1670 University Blvd, Birmingham, USA

**Keywords:** ^89^Zr, MMTV-HER2, 4T1, PD-1, CTLA4, Spleen, Positron emission tomography, ImmunoPET, Spatial heterogeneity

## Abstract

**Background:**

Immune-positron emission tomography (PET) imaging with tracers that target CD8 and granzyme B has shown promise in predicting the therapeutic response following immune checkpoint blockade (ICB) in immunologically “hot” tumors. However, immune dynamics in the low T-cell infiltrating “cold” tumor immune microenvironment during ICB remain poorly understood. This study uses molecular imaging to evaluate changes in CD4 + T cells and CD8 + T cells during ICB in breast cancer models and examines biomarkers of response.

**Methods:**

[^89^Zr]Zr-DFO-CD4 and [^89^Zr]Zr-DFO-CD8 radiotracers were used to quantify changes in intratumoral and splenic CD4 T cells and CD8 T cells in response to ICB treatment in 4T1 and MMTV-HER2 mouse models, which represent immunologically “cold” tumors. A correlation between PET quantification metrics and long-term anti-tumor response was observed. Further biological validation was obtained by autoradiography and immunofluorescence.

**Results:**

Following ICB treatment, an increase in the CD8-specific PET signal was observed within 6 days, and an increase in the CD4-specific PET signal was observed within 2 days in tumors that eventually responded to immunotherapy, while no significant differences in CD4 or CD8 were found at the baseline of treatment that differentiated responders from nonresponders. Furthermore, mice whose tumors responded to ICB had a lower CD8 PET signal in the spleen and a higher CD4 PET signal in the spleen compared to non-responders. Intratumoral spatial heterogeneity of the CD8 and CD4-specific PET signals was lower in responders compared to non-responders. Finally, PET imaging, autoradiography, and immunofluorescence signals were correlated when comparing in vivo imaging to ex vivo validations.

**Conclusions:**

CD4- and CD8-specific immuno-PET imaging can be used to characterize the in vivo distribution of CD4 + and CD8 + T cells in response to immune checkpoint blockade. Imaging metrics that describe the overall levels and distribution of CD8 + T cells and CD4 + T cells can provide insight into immunological alterations, predict biomarkers of response to immunotherapy, and guide clinical decision-making in those tumors where the kinetics of the response differ.

**Supplementary Information:**

The online version contains supplementary material available at 10.1186/s13058-024-01844-3.

## Background

Immune checkpoint blockade (ICB) is used clinically to treat a diverse range of cancers, including breast cancer [1, 2]. The efficacy of ICB in breast cancer varies greatly, with only a small percentage of patients experiencing durable and impactful responses [3]. Recent studies have highlighted the important role of tumor-infiltrating lymphocytes (TILs), specifically CD8 + cytotoxic T cells and CD4 + T cells, in the ICB treatment response [4, 5]. Therefore, monitoring of TIL populations, their distribution, and activation [6] can help predict the efficacy of ICB treatment [7] and understand variations in the anti-tumor response. Non-invasive positron emission tomography (PET) imaging that targets TILs has shown promise in predicting clinical outcomes of ICB, with increased accumulation of CD8 + T cells as a predictor of tumor regression [8]. While CD8 + T cells have been a main focus in the development of predictive markers, there is mounting evidence that highlights the role of CD4 + T cells in the efficacy of ICB [9]. PD-1 blockade targets exhausted intratumoral CD4 + cells and restores their helper activity [10]. This results in a systemically elevated CD4 T cell population, linked to better clinical efficacy in PD-L1/PD-1 blockade therapies [11]. However, the potential of PET imaging to target CD4 + T cells as a predictor of the response to ICB and monitor the efficacy of treatment remains to be shown.

Breast cancer is traditionally considered an immunologically “cold” tumor, with limited T cell infiltration [2]. Some subtypes of breast cancer, such as triple-negative breast cancer (TNBC), show relatively large numbers of TILs; however, overall response rates to ICB in TNBC remain low [12]. In unselected TNBC patients, the response rates of anti-PD1 treatment are 10% and only slightly increase to 20–30% when patients are selected based on immunohistochemistry (IHC)-confirmed expression of PD-L1+ [12]. Current preclinical studies have shown a strong relationship between the presence or the activation of TILs (e.g., Granzyme B level), evaluated via PET imaging (e.g., GZP-PET [6]), and ICB efficacy. However, these studies have mostly concentrated on models characterized by an immunologically “hot” tumor microenvironment, such as colon cancer [6, 13], defined by the presence of large numbers of TILs. Many immunologically “cold” tumors will be selected in a clinical setting as candidates for immunotherapy. Therefore, it is important to understand how monitoring longitudinal changes in TILs utilizing PET imaging in immunologically “cold” tumors can predict the efficacy of ICB treatment.

Intratumoral and systemic numbers of immune cell populations and levels of protein expression are typically determined by tumor biopsies or sampling of peripheral blood [14]. These more conventional assays have shown wide variations in the immune profiles of tumors post-therapy with transient changes in the numbers of CD8 + and CD4 + T cells [4, 7]. PET imaging provides a powerful, non-invasive method to measure cell metabolism, cell proliferation, or protein expression in real-time [15–17]. PET imaging of the entire animal can reduce the bias of spatial heterogeneity inherent in biopsy samples. Longitudinal monitoring of immune cell trafficking and infiltration can provide information on both the presence of T cells at baseline and on the immunological changes in response to therapy. ImmunoPET imaging of CD4 + and CD8 + T cells can quantify the relationship between the response to ICB and intratumoral heterogeneity in the distribution of TILs. Previous studies have established a correlation between the intratumoral heterogeneity of CD8 T cell distribution and response to ICB in immunologically “hot” tumors, with a heterogeneous distribution of PET signal within the tumor considered an indicator of poor outcomes [18]. However, considering the low levels of TILs in immunologically “cold” tumors, the question remains of whether spatial heterogeneity in the intratumoral distribution of CD4 + and CD8 + T cells can be a useful indicator to predict the efficacy of ICB treatment.

This study investigates the potential of non-invasive immunoPET imaging to be used as a predictive tool to assess the response to ICB treatment in immunologically “cold” breast tumors. By assessing the dynamics and heterogeneity in the distribution of CD4 + and CD8 + TILs, we aim to study the relationship between TILs and treatment outcomes. This research should help improve patient selection as well as the development of personalized approaches for ICB therapy in breast cancer.

## Materials and methods

### Cell culture and mouse models

4T1 cells were purchased from ATCC (catalog number: CRL-2539-LUC2) and cultured in Roswell Park Memorial Institute (RPMI) 1640 Medium (Fisher, 11-875-119) with 10% (v/v) Fetal Bovine Serum (FBS) and 1% (v/v) L-glutamine. 2 × 10^5^ 4T1 cells were injected in the 1st mammary fat pad of 6-week-old female Balb/c mice (*N* = 80). MMTV-HER2 tumors (*N* = 80) were generated as previously reported [19]. Fresh tumor chunks (2–3 pieces per mouse at 1mm^3^) in 50µL Matrigel were transplanted into the 1st mammary fat pad of FVB mice (female, 5–6 weeks old, implanted with estrogen pellet 24 h before tumor implantation). Tumor volumes were measured every 2–3 days with a caliper. Tumor volumes were calculated using the formula: $V=\frac{1}{6}{\pi \left(\frac{short diameter}{2}\right)}^{2}\left(long diameter\right)$. Animals entered the experiment when their tumor volume reached 50–150 mm^3^. Saline (*N*=4 per group), α-PD1 (InVivoMab, BE0146; 200 µg per mouse, *N*=12 per group), α-CTLA4 (InVivoMab, BE0164; 200 µg per mouse, *N* = 12 per group) or the combination of α-PD1 and α-CTLA4 (*N* = 12 per group) were i.v. injected at treatment days 0, 3, and 6. Based on the tumor mass at the final time point, tumors were assigned to the responder or non-responder groups using the control tumor mass as a threshold. The threshold for responding tumors was defined as tumors with a tumor mass less than the control tumors’ mean mass minus the standard deviation (< mean-SD) at the terminal time point. All animal housing and procedures were conducted under the guidelines provided by the Institutional Animal Care and Use Committee of The University of Alabama at Birmingham.

### [^89^Zr]Zr-DFO-CD4 and [^89^Zr]Zr-DFO-CD8 PET/ computed tomography (CT) imaging

Mouse anti-CD4 (Df-IAB46; ImaginAb) and anti-CD8 (Df-IAB42; ImaginAb) minibodies conjugated with deferoxamine (DFO) chelators were labeled with [^89^Zr] at 0.37 MBq/µg at 37^o^C for 1 h (pH 6.8–7.2). The radiochemical yield (RCY) was assessed via instant thin-layer chromatography (iTLC), with 50 mM DTPA serving as the developing solution. RCYs ≥ 95% in chromatography were deemed suitable for in vivo studies without additional purification. For yields lower than 95%, the radiotracer underwent purification using Zeba spin desalting columns (Thermo Scientific, 89,877) to attain radiochemical purity (RCP) ≥ 95%. For long-term experiments, 3.7 ± 0.19 MBq (10 ± 0.5 µg) of radiotracer was injected into mice via the retro-orbital sinus with static PET scans conducted at 24 h, 72 h, and 7 days post-injection with a preclinical GNEXT small animal PET/CT machine (Sofie Biosciences, Dulles, VA) for 20, 30, 40 min, respectively. For blocking, biodistribution, and autoradiography experiments, 1.85 ± 0.19 MBq (5 ± 0.5 µg) of radiotracer was injected intravenously and PET imaging was conducted 24 h post-injection. A CT scan was acquired following the acquisition of the PET signal to obtain an anatomical reference. Regions of interest (ROIs) around the tumor, contralateral muscle, heart, and spleen were drawn manually using the CT scan for guidance, and data was analyzed with VivoQuant software (Invicro, Boston, MA). The standard uptake value was calculated using $SUV=\frac{C}{dose/weight}$, where $C$ was defined as the tissue radioactivity concentration, dose as the administered dose calibrated to imaging time for isotope decay (all images were acquired at the same settings), and weight as the mouse body weight. The SUV_mean_ represents the average SUV within the ROI, while the SUV_peak_ denotes the maximum average SUV within a small, fixed-size ROI consisting of 27 voxels. Quantification of tissue heterogeneity was performed by identifying regions of peak concentration from the tumor PET signal with an automated MATLAB R2022 (Mathworks, Natick, MA) adopted from previously published work [20]. A mask of regional peaks for each tumor PET slice was identified and the numbers of peaks were quantified as 3D volumetric objects to provide a quantitative approach to assess spatial heterogeneity (code available upon request).

### Blocking and biodistribution experiments

FVB mice (*N* = 16) were randomly assigned into four groups: CD4 imaging agent, CD4 imaging agent + blocking, CD8 imaging agent, and CD8 imaging agent + blocking. Each mouse received an i.v. injection of ∼ 5 µg of radiolabeled CD4 or CD8 minibody and underwent PET imaging 24 h later. 50 µg of unlabeled CD4 or CD8 minibody was used as a blocking agent and co-injected with the radiotracer. Organs and blood were collected immediately after PET for biodistribution.

### Autoradiography

4T1 tumor-bearing Balb/c mice were randomly assigned into four groups: CD4-PET with saline treatment, CD4-PET with α-PD1 + α-CTLA4 treatment, CD8-PET with saline treatment, and CD8-PET with α-PD1 + α-CTLA4 treatment. Mice received treatments (saline or 200 µg α-PD1 + 200 µg α-CTLA4) via i.v. injection at 7-, 10-, and 13-days post-tumor injection. Radiotracer was injected at 13 days post-tumor injection. PET imaging and tissue collection were performed 24 h post-radiotracer injection. Tumors were fixed in 10% formalin overnight, cut into 1 mm-thick slices, and subjected to autoradiography with film exposure for three hours and image acquisition using the Typhoon Biomolecular Imager. Quantification of radiotracer uptake in tissue was quantified with 1 nCi, 2 nCi, and 10 nCi radiotracer standards for reference (VivoQuant software, Invicro, Boston, MA).

### Immunofluorescence (IF) staining

IF staining was performed as described [21]. Briefly, paraffin-embedded tumors were sectioned as 5 μm slides and dewaxed with xylene. Citrate buffer (Abcam, ab93678) was used for antigen retrieval. Tissues were blocked with 1% BSA and 0.02% milk in PBS with 0.03% Triton X100 for 1 h at room temperature. Primary antibody anti-CD4 (Fisher, BDB553647) or anti-CD8 (Fisher, BDB558733) were applied overnight at 4^o^C. Fluorescein isothiocyanate (FITC) conjugated donkey anti-rat-IgG antibody was used as a secondary antibody and incubated for 1 h at room temperature. Finally, slides were mounted with DAPI mounting media (SouthernBiotech, 0100 − 20). High-resolution 20x images were acquired (EVOS M7000 Imaging System, Thermo Fisher Scientific, Waltham, MA) and CD4 + or CD8 + cells were quantified as previously reported (MATLAB) [21].

### Statical analysis

**Fig. 1:**
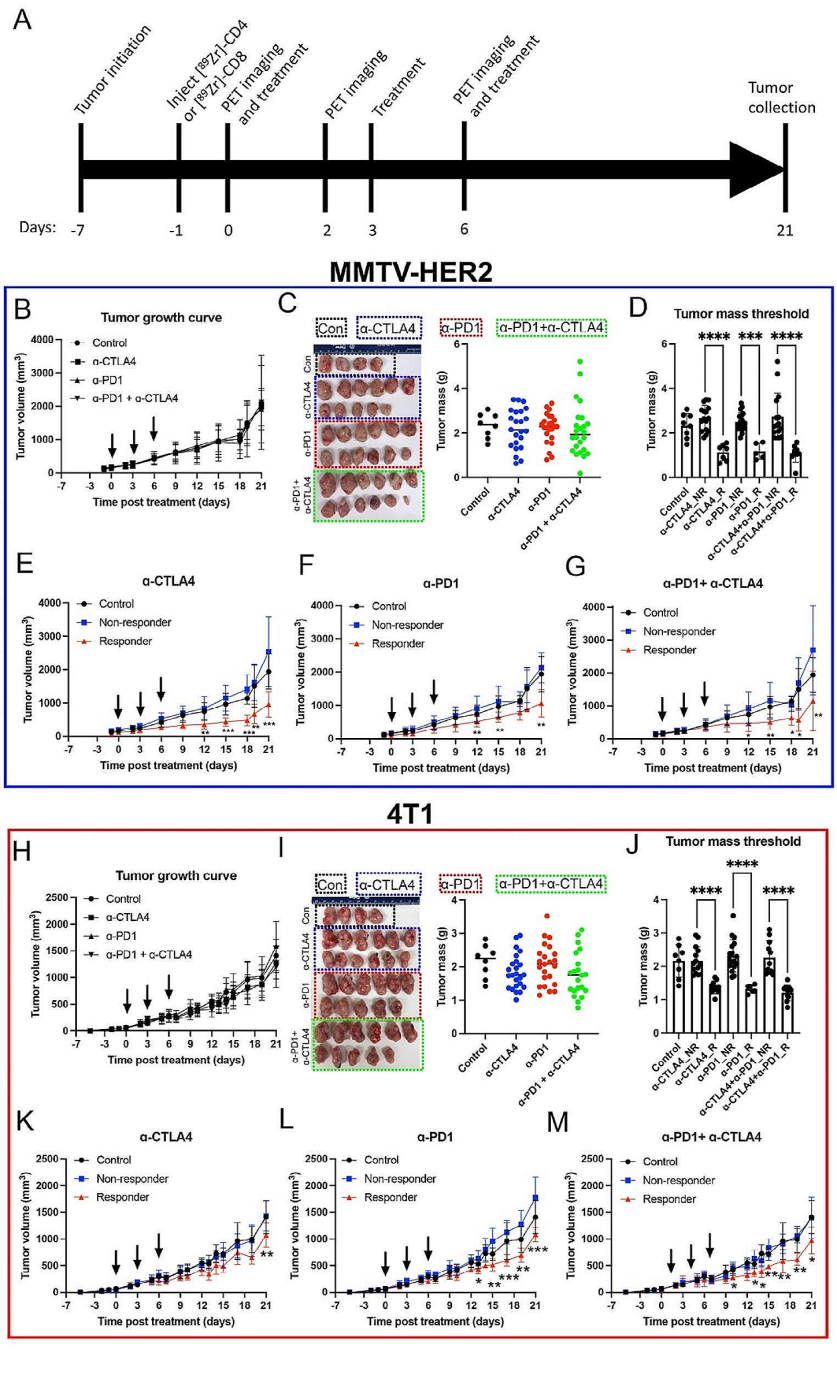
ICB treatment response varies in MMTV-HER2 and 4T1 mouse models. A) Experimental timeline. **B**) Tumor growth curve of MMTV-HER2 model. **C**) Image of tumors at the end of the experiment in the MMTV-HER2 model. The combinational treatment group showed a trend of decrease in tumor mass, yet the decrease was not significant. **D**) Tumor mass of threshold method in MMTV-HER2 model. **E-F**) Tumor growth curve after thresholding in MMTV-HER2 model. Responders showed significantly decreased tumor volumes. **H**) Tumor growth curve of 4T1 model. **I**) Image of tumors at the end of the experiment in the 4T1 model. **J**) Tumor mass of threshold method in the 4T1 model. **K-M**) Tumor growth curve after thresholding in the 4T1 model. *N* = 80. R was for responders. NR was for non-responders

**Fig. 2:**
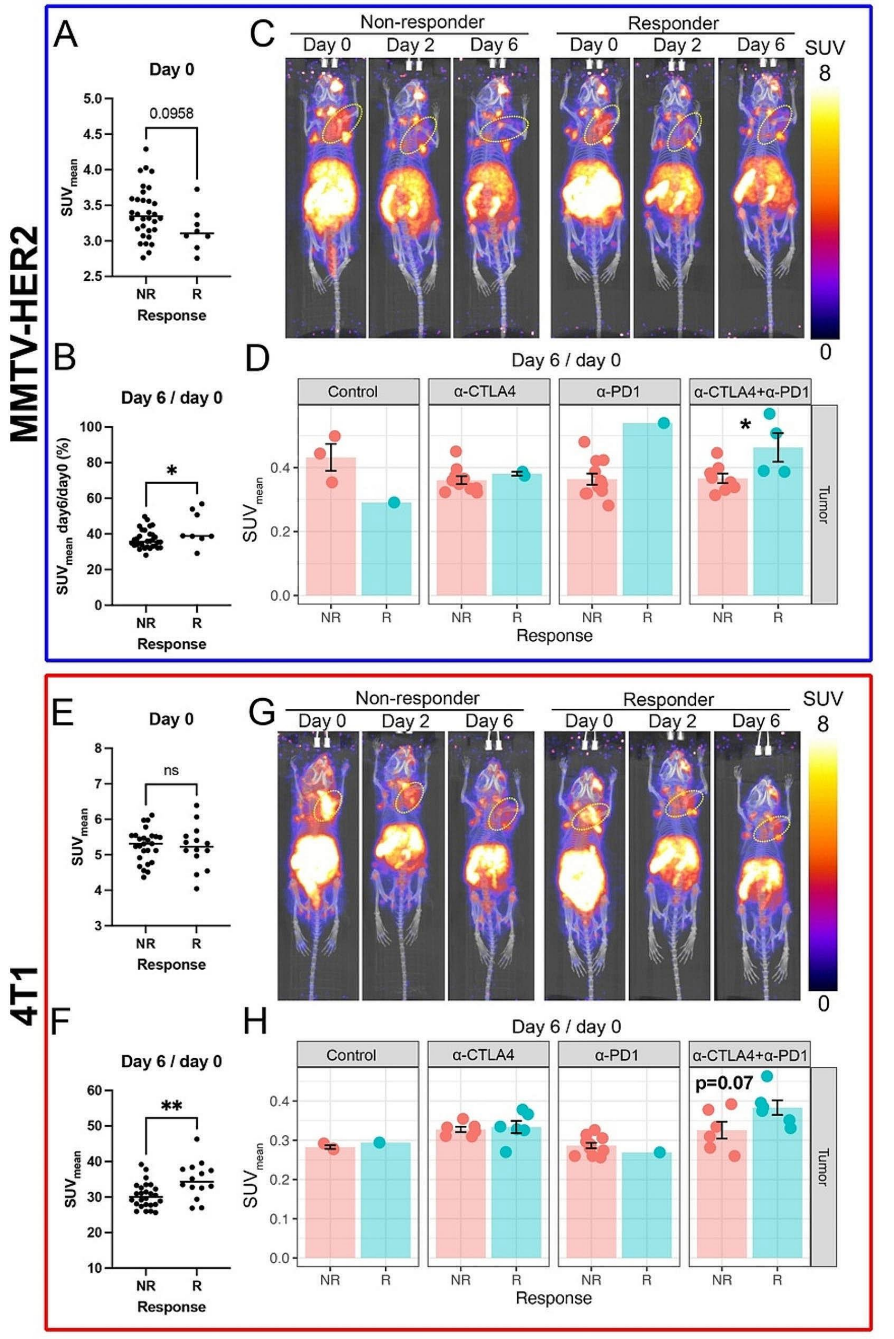
Intratumoral CD8-specific PET signals, [^89^Zr]Zr-DFO-CD8 SUV_mean_, increased in tumors that had an effective response to ICB. **A**) The mean of standard uptake value (SUV_mean_) of responders showed a decreasing trend in the MMTV-HER2 model at day 0. **B**) From day 0 to 6, changes in SUV_mean_ of responders showed an increase compared to non-responders in the MMTV-HER2 model. Overall, CD8 + cells were retained/infiltrated from day 0 to day 6 in MMTV-HER2 tumors that responded to ICB. **C**) Representative images of [^89^Zr]Zr-DFO-CD8 PET from day 0 to day 6 in the combinational treatment group in the MMTV-HER2 model. Yellow circles indicated tumors. All the images are processed at the same setting and decay corrected to the imaging time. **D**) Day6/day0 intratumoral [^89^Zr]Zr-DFO-CD8 SUV_mean_ of each treatment group in MMTV-HER2 model. In the combinational treatment group, responders had an increase of [^89^Zr]Zr-DFO-CD8 SUV_mean_ from day 0 to day 6 compared to non-responders. **E**) [^89^Zr]Zr-DFO-CD8 SUV_mean_ at day 0 in 4T1 model. No significance was found. **F**) The ratio of day6/day0 of [^89^Zr]Zr-DFO-CD8 SUV_mean_ in 4T1 model. Overall, CD8 + cells were retained/infiltrated from day 0 to day 6 in 4T1 tumors that responded to ICB. **G**) Representative images of [^89^Zr]Zr-DFO-CD8 PET from day 0 to day 6 in the combinational treatment group in the 4T1 model. Yellow circles indicated tumors. All the images are processed at the same setting and decay corrected to the imaging time. **H**) Day6/day0 intratumoral [^89^Zr]Zr-DFO-CD8 SUV_mean_ of each treatment group in the 4T1 model

**Fig. 3:**
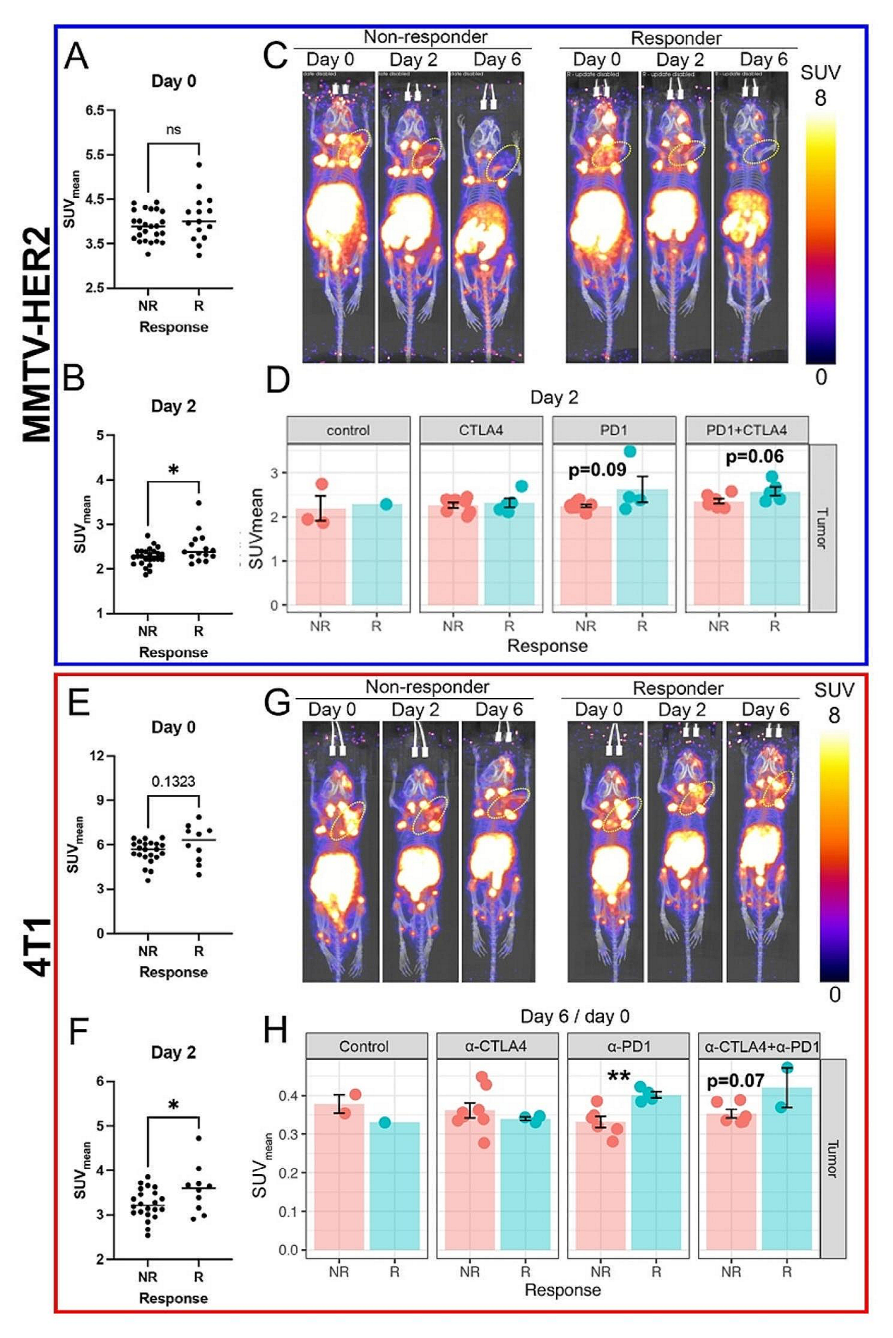
Intratumoral CD4-specific PET signals, [^89^Zr]Zr-DFO-CD4 SUV_mean_, on day 2 increased in ICB responders. A-B) Mean of standard uptake value (SUV_mean_) at day 0 (**A**) and 2 (**B**) in MMTV-HER2 model. Overall, CD4 + cells were increased in the retained/infiltrated population in ICB responders in MMTV-HER2 model. **C**) Representative images of [^89^Zr]Zr-DFO-CD4 PET from day 0 to day 6 in the combinational treatment group in the MMTV-HER2 model. Yellow circles indicated tumors. The responders showed a slightly increased [^89^Zr]Zr-DFO-CD4 signal on day 2. All the images are processed at the same setting and decay corrected to the imaging time. **D**) Day 2 intratumoral [^89^Zr]Zr-DFO-CD4 SUV_mean_ of each treatment group in MMTV-HER2 model. There was a trend of increase in α-PD-1 treated responders in the MMTV-HER2 model. **E-F**) [^89^Zr]Zr-DFO-CD4 SUV_mean_ at day 0 (**E**) and 2 (**F**) in the 4T1 model. On day 2, retained/infiltrated CD4 + cells increased in ICB treatment responders relative to non-responders. **E**) Representative images of [^89^Zr]Zr-DFO-CD4 PET from day 0 to day 6 in the combinational treatment group in the 4T1 model. Yellow circles indicated tumors. The responder group showed a slightly increased [^89^Zr]Zr-DFO-CD4 signal on day 2. All the images are processed at the same setting and decay corrected to the imaging time. **F**) Day6/day0 intratumoral [^89^Zr]Zr-DFO-CD4 SUV_mean_ of each treatment group in the 4T1 model. α-PD-1 treated responders showed a significant increase of [^89^Zr]Zr-DFO-CD4 SUV_mean_. **G**) Representative images of [^89^Zr]-CD4 PET from day 0 to day 6 in the combinational treatment group in the 4T1 model. The responder showed a slight increase in [^89^Zr]-CD4 signal on day 2. **H**) Day6/day0 intratumoral [89Zr]-CD4 SUV_mean_ of each treatment group in 4T1 model. α-PD-1 treated responders showed a significant increase of [^89^Zr]-CD4 SUV_mean_

**Fig. 4:**
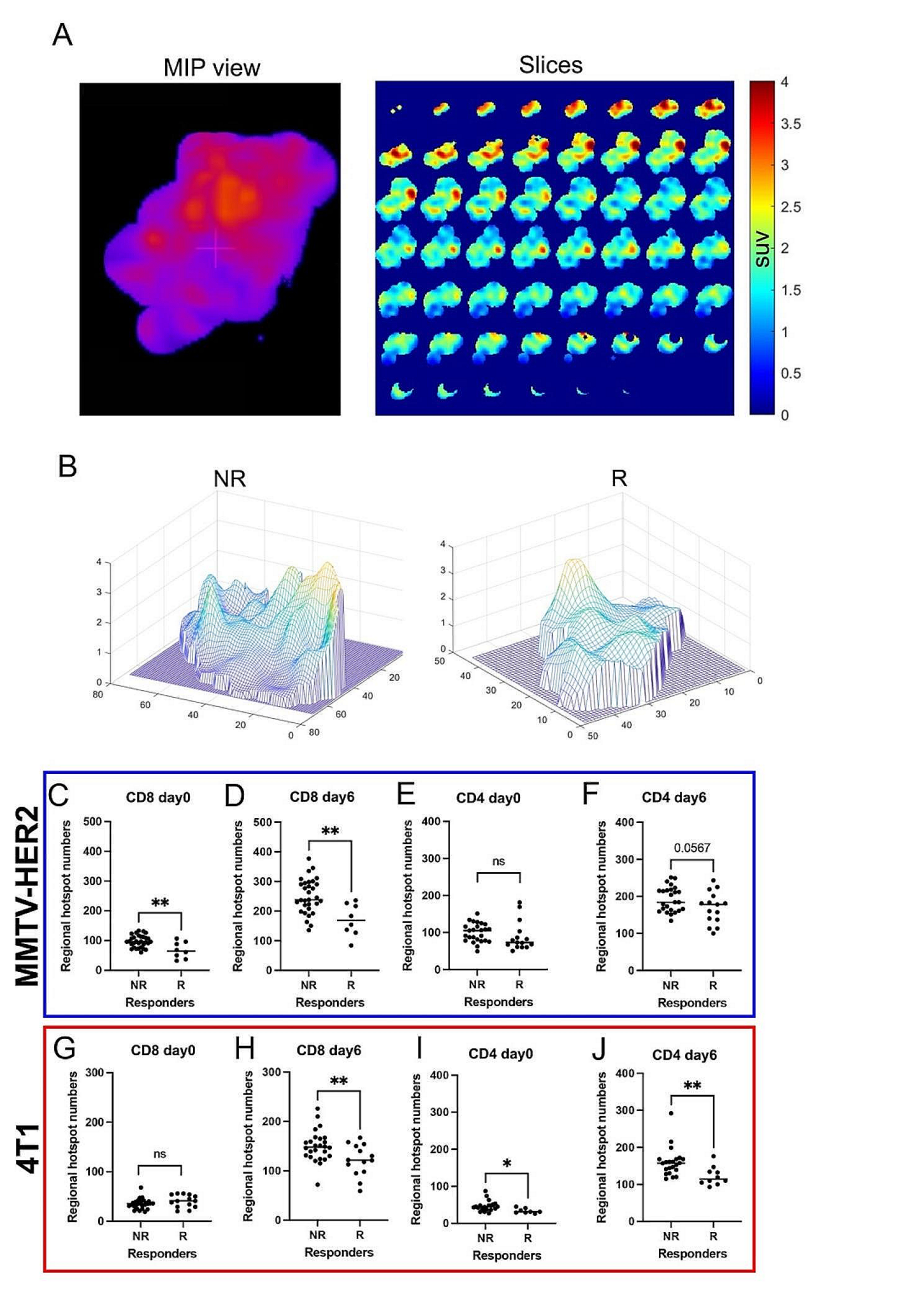
The spatial heterogeneity of intratumoral CD4 and CD8 PET signals was reduced in responders. **A**) Representative images of isolated 3D tumors and 2D slices of PET image. **B**) Representative topographic map of 2D slice PET images. In the MMTV-HER2 model, non-responders showed increased peak numbers of [^89^Zr]Zr-DFO-CD8 uptake compared to responders. **C-J**) Quantification of regional hotspot numbers in 3D tumors. In both MMTV-HER2 and 4T1 models, the responders showed decreased regional hotspot numbers of [^89^Zr]Zr-DFO-CD8 and [^89^Zr]Zr-DFO-CD4 uptake compared to non-responders after initiation of ICB (day 6)

**Fig. 5:**
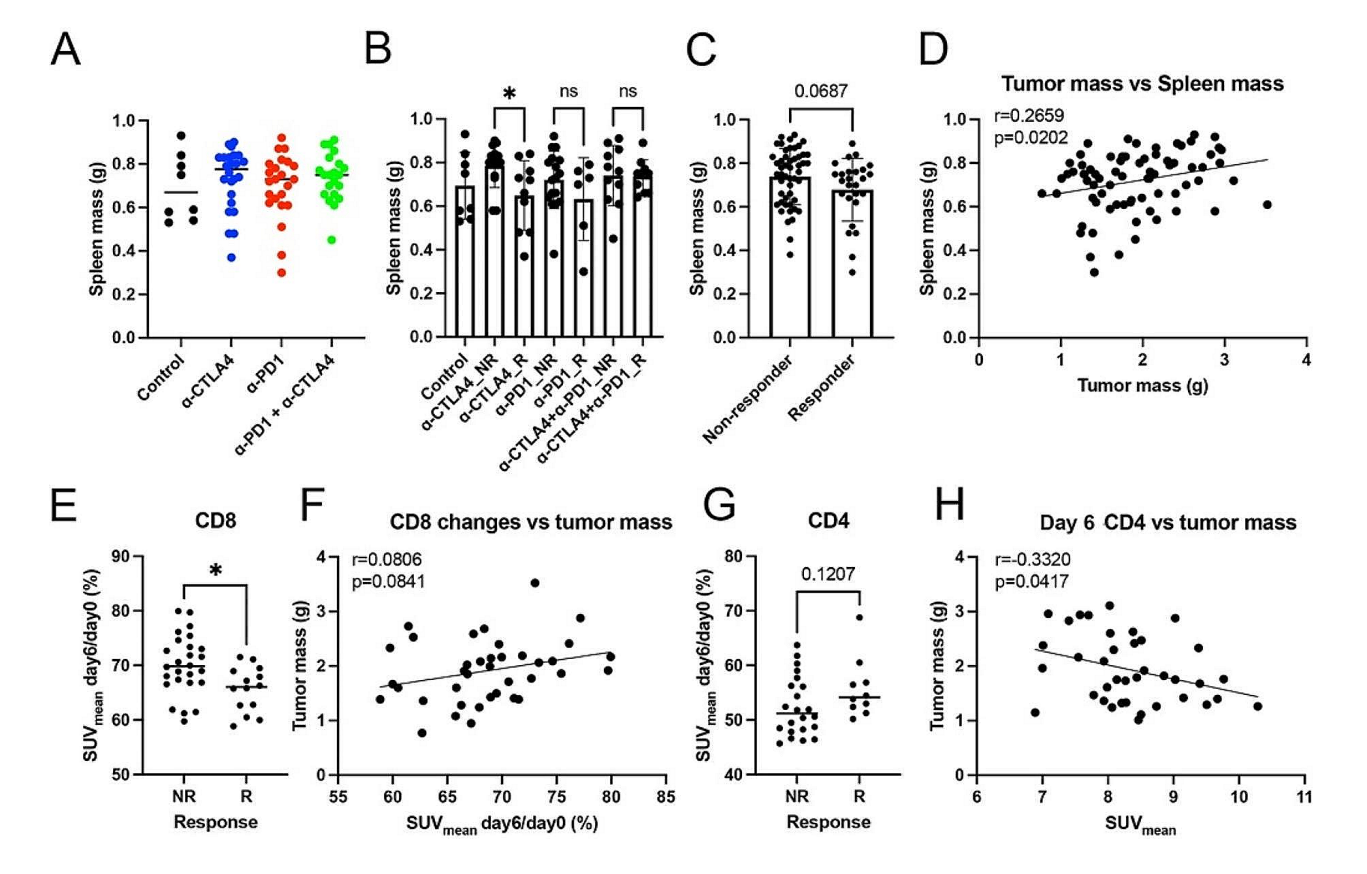
The CD8-specific PET signal in the spleen was decreased in responders and the CD4-specific PET signal was positively correlated with the efficacy of ICB treatment in 4T1 models. A-C) Terminal spleen mass in different ICB treatment groups and responses. ICB (especially α-CTLA4) treatment responders showed decreased spleen mass. **D**) There was a significant correlation between terminal tumor mass and spleen mass (*r* = 0.2659, *p* = 0.0202). **E**) There was a significant decrease in splenic [^89^Zr]Zr-DFO-CD8 in ICB responders compared to non-responders. **F**) Dynamic changes from day 0 to day 6 of splenic [^89^Zr]Zr-DFO-CD8 SUV_mean_ showed a weak trend of correlation with terminal tumor mass (*r* = 0.0806, *p* = 0.0841). The less retained splenic CD8 + cells indicated a smaller terminal tumor mass and a bigger terminal spleen mass. **G**) There was a trend of an increase in splenic [^89^Zr]Zr-DFO-CD4 in ICB responders compared to non-responders. **H**) There was a significant correlation between day 6 [^89^Zr]Zr-DFO-CD4 SUV_mean_ and terminal tumor mass (*r* = 0.3320, *p* = 0.0417). The higher CD4 signal in the spleen on treatment day 6 favored a better treatment outcome

Unpaired T test was used in Figs. 2, 3 and 4C, K, E-F and M-N, and 5. One-way ANOVA with Bonferroni correction was used in Figs. 1D and J and 4B, and 4J. Two-way ANOVA with Tukey post hoc test was used in Fig. 1E-G and K-M, and the Pearson correlation test was used in Fig. 4D, G-H and L, and 4O-P. non-significance (ns), *p* > 0.05; *, *p* < 0.05; **, *p* < 0.01; ***, *p* < 0.001.

## Results

### “Cold” tumors showed a variable response to ICB therapy

To examine the response to ICB in “cold” tumors, MMTV-HER2 (HER2 + breast cancer) and 4T1 (TNBC) tumors were treated with α-PD1, α-CTLA4, or a combination. Both models have relatively few infiltrating T cells in the naïve tumor microenvironment [22, 23] and respond similarly [22] to ICB in breast cancer patients as clinically documented (clinical benefit rate of ∼ 20%) [24]. The experimental timeline is depicted in Fig. 1A. Tumor growth curves (MMTV-HER2, Fig. 1B; **4T**1, Fig. 1H) showed no significant differences between treatment groups. However, a heterogeneous departure from the baseline was seen, particularly in the ICB-treated groups. The tumor mass at necropsy varied significantly in the ICB-treated groups, with the highest variations in the combination treatment group (Fig. 1C, I). Mice in the ICB treatment groups were categorized as non-responders and partial responders (referred to as responders thereafter) based on tumor mass thresholding at the terminal time point (see the thresholding in the methods section) (Fig. 1D, J). Stratifying responders and non-responders based on terminal tumor mass led to significant distinctions in tumor volumes for each ICB treatment group (Fig. 1E-G, K-M, *p* < 0.05).

### Changes in the composition of intratumoral CD8 + and CD4 + T cells in the immune microenvironment in “cold” tumors showed in response to ICB

To evaluate changes in the composition of intratumoral CD8 + and CD4 + cells over time during ICB treatment, longitudinal [^89^Zr]Zr-DFO-CD8, and [^89^Zr]Zr-DFO-CD4 PET imaging was performed. The specificity of [^89^Zr]Zr-DFO-CD8 and [^89^Zr]Zr-DFO-CD4 PET imaging was validated by blocking experiments (Supplemental Results and Supplemental Fig. 1). In the MMTV-HER2 model, baseline (day 0) intratumoral [^89^Zr]Zr-DFO-CD8 SUV_mean_ (Fig. 2A, *p* = 0.0958) and SUV_peak_ (Supplemental Fig. 2C, p = 0.0619) showed a decreasing trend in the responder group compared to the non-responder group. In the 4T1 model, baseline [^89^Zr]Zr-DFO-CD8 uptake showed no significant differences between responders and non-responders (Fig. 2E, Supplemental Fig. 2F, p > 0.05). Overall, in both models, the ratio of day 6 to day 0 of SUV_mean_ in responders was significantly higher than that in non-responders (Fig. 2B, *p* < 0.05; **2F**, *p* < 0.01). Further, the SUV_peak_ was reported to be the most reliable parameter for [^18^F]FDG-PET quantification in avid glucose tumors [25]. In our study, SUV_peak_ did not show a significant difference Supplemental Fig. 2C & 2F, p > 0.05), demonstrating the need to incorporate information from the entire tumor. Compared to non-responders, responder groups showed an increase in the retention of [^89^Zr]Zr-DFO-CD8 in both MMTV-HER2 (Fig. 2C) and 4T1 models (Fig. 2G). For both tumor models, the combination of α-PD-1 and α-CTLA4 resulted in the most significant differences in CD8 signal when comparing responders and non-responders. Within the combinational treatment group, there was a significant increase in CD8-specific PET signal in the MMTV-HER2 model (Fig. 2D, *p* < 0.05) and a noticeable trend (increase) in the 4T1 model that did not reach significance (Fig. 2H, *p* = 0.07) when comparing the ratio of day 6 to day 0.

Early during ICB treatment (day 2, following one dose of ICB), uptake of the CD4 tracer (measured by [^89^Zr]Zr-DFO-CD4 SUV_mean_) was significantly increased in the responders compared to non-responders in both models (Fig. 3B, *p* < 0.05; **3 F**, *p* < 0.05). However, changes of intratumoral [^89^Zr]Zr-DFO-CD4 SUV_mean_ showed no significant differences between responders and non-responders in both MMTV-HER2 (Fig. 3A, Supplemental Fig. 3A, p > 0.05) and 4T1 models (Fig. 3E, Supplemental Fig. 3D, p > 0.05). Intratumoral [^89^Zr]Zr-DFO-CD4 SUV_peak_ showed trending increases in the responder tumors in the MMTV-HER2 model on day 6, but these did not reach statistical significance (Supplemental Fig. 3B, p = 0.09). There was a significantly higher [^89^Zr]Zr-DFO-CD4 SUV_peak_ in the responding group in the 4T1 model at baseline (Supplemental Fig. 3E, p < 0.05). Further, there was increased retention of [^89^Zr]Zr-DFO-CD4 on day 2 in responder groups for both the MMTV-HER2 (Fig. 3C) and 4T1 models (Fig. 3G), as shown in representative images. For each treatment condition, α-PD1 treatment showed a significant increase in intratumoral [^89^Zr]Zr-DFO-CD4 SUV_peak_ on day 2 in the MMTV-HER2 model (Supplemental Fig. 3G, p < 0.05), and in the ratio of day 6 to day 0 of [^89^Zr]Zr-DFO-CD4 SUV_mean_ in the 4T1 model (Fig. 3H, *p* < 0.01). These findings suggest that α-PD1 treatment played an important role in the intratumoral CD4 + cell population.

Taken together, the intratumoral changes over time of CD8-specific PET signal, measured with PET imaging, showed that increases in retention of CD8 T cells in the tumor were positively correlated with the efficacy of ICB treatment. Similarly, the analysis of the CD4 population showed that a higher intratumoral presence of CD4 T cells two days after one ICB dose was indicative of better ICB response.

### ICB responders had low spatial heterogeneity of CD4 + and CD8 + T cells in the intratumoral microenvironment

Intratumoral spatial heterogeneity of PET signals was quantified (an example of image processing was shown in Fig. 4A) to assess how the spatial distribution of CD4 + and CD8 + T cells affects the response to ICB treatment in “cold” tumors. Representative cross-sectional PET images of [^89^Zr]Zr-DFO-CD8 PET at day 6 in the MMTV-HER2 model showed that the non-responders had more variation in the number of hotspots (high SUV spots) compared to responders (Fig. 4B). In the quantification of the 3D PET images, responding tumors had significantly fewer [^89^Zr]Zr-DFO-CD8 hotspots than non-responders in both MMTV-HER2 (Fig. 4D, *p* < 0.01) and 4T1 model on day 6 (Fig. 4H, *p* < 0.01). Regarding CD4 + T cells, responders had fewer [^89^Zr]Zr-DFO-CD4 SUV hotspots in the 4T1 model (Fig. 4J, *p* < 0.01) and in the MMTV-HER2 tumors (Fig. 4F, *p* = 0.057) on day 6 compared to non-responders. Moreover, baseline heterogeneity in the responding cohort was significantly lower in MMTV-HER2 tumors with [^89^Zr]Zr-DFO-CD8 (Fig. 4C, *p* < 0.01) and 4T1 models with [^89^Zr]Zr-DFO-CD4 (Fig. 4I, *p* < 0.05). Alterations from day 0 to day 6 and day 2 heterogeneity analysis are seen in supplementary Fig. 4. Overall, the decrease in regional hotspots ([^89^Zr]Zr-DFO-CD8 and [^89^Zr]Zr-DFO-CD4) after treatment indicated better control of tumor burden.

### Changes in the immune microenvironment of “cold” tumors correlate with a decrease in splenic CD8 + T cells and an expansion of CD4 + T cells

To evaluate systemic changes in the immune microenvironment of “cold” tumors, splenic [^89^Zr]Zr-DFO-CD8 and [^89^Zr]Zr-DFO-CD4 uptake and terminal spleen mass were quantified. In the MMTV-HER2 model, spleen mass was not significantly different across treatment groups (Supplemental Fig. 5A-B, p > 0.05). Interestingly, a significantly lower spleen mass was observed in tumors responsive to CTLA4 compared to non-responders (Fig. 5A-C, *p* < 0.05). Terminal tumor and spleen mass were positively correlated in the 4T1 model (Fig. 5D, *r* = 0.27, *p* = 0.02), suggesting there may be splenic components that play important roles in ICB response. Responding tumors showed a significantly decreased SUV_mean_ (ratio of day 6 to day 0) of [^89^Zr]Zr-DFO-CD8 compared to non-responders in the 4T1 model (Fig. 5E, *p* < 0.05). The observed increase in intratumoral CD8 signal and the corresponding decrease in splenic CD8 signal, as evidenced by the post-to-pre-treatment ratio, suggest potential trafficking of CD8 + cells from the spleen to the tumor site. Further, splenic CD4 signal (SUV_mean_ on day 6) was negatively correlated with terminal tumor mass (Fig. 5H, *r* = 0.33, *p* = 0.04) in the 4T1 model, indicating that higher spleen CD4 + cell presence was related to smaller tumor sizes. These results indicate that decreased splenic CD8 + cells and increased splenic CD4 + cells are associated with favored ICB treatment outcomes.

### Autoradiography and immunofluorescence (IF) staining validated the in vivo PET signals for CD8 + and CD4 + T cells. Spatial heterogeneity and overall uptake are distinct measurements

**Fig. 6:**
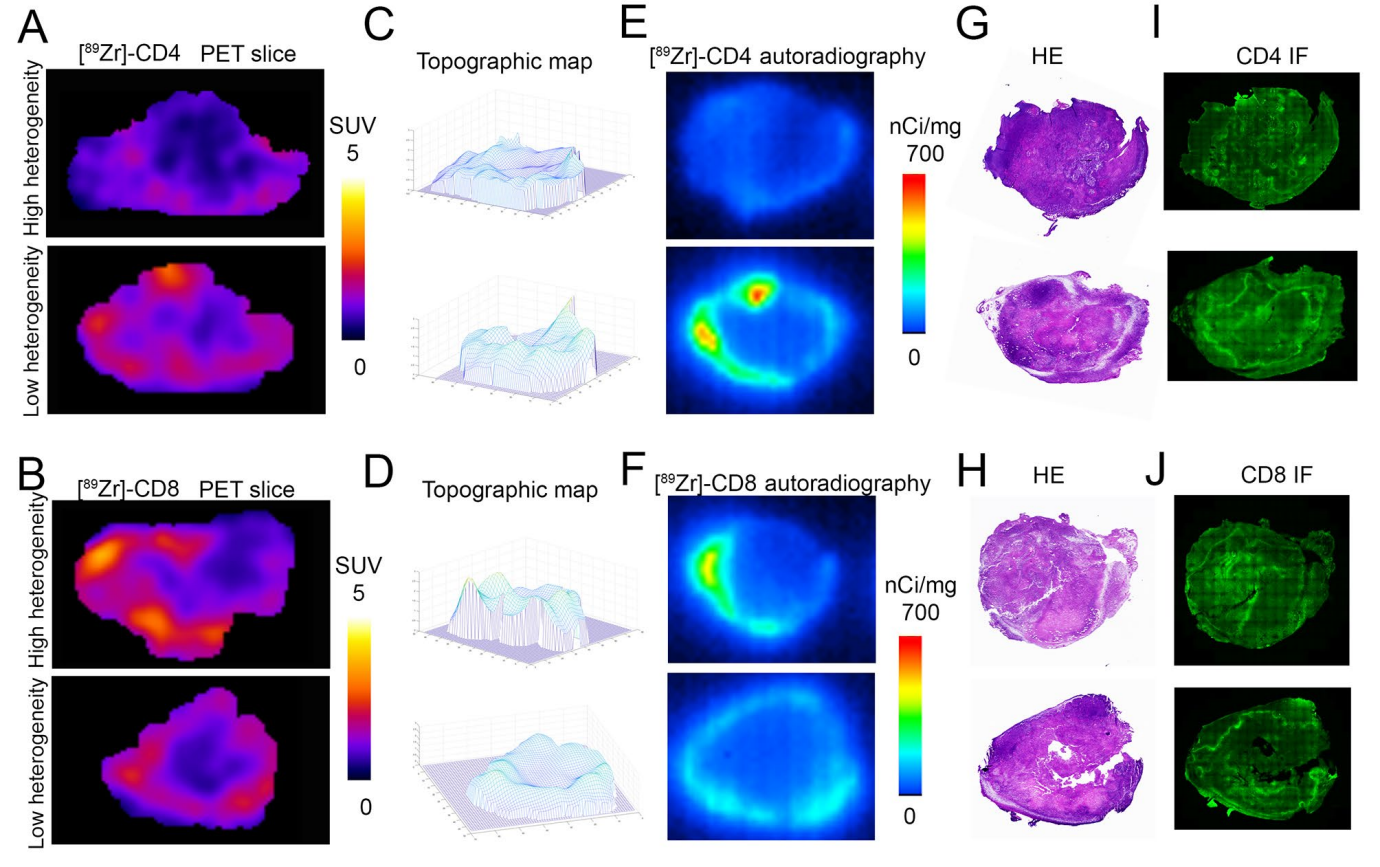
Autoradiography and immunofluorescence (IF) staining show that spatial heterogeneity and overall uptake are distinct measurements. **A-B**) Representative central slice of PET images showed high and low heterogeneity of [^89^Zr]Zr-DFO-CD4 and [^89^Zr]Zr-DFO-CD8 distribution, despite differences in total levels of uptake. **C-D**) Topographic map of [^89^Zr]Zr-DFO-CD4 and [^89^Zr]Zr-DFO-CD8 PET slices indicated regional hotspots. **E-F**) Corresponding autoradiography images immediately after PET imaging. **G-H**) Hematoxylin and eosin (H&E) staining of 4T1 tumor sections revealed that areas of high [^89^Zr]Zr-DFO-CD4 or [^89^Zr]Zr-DFO-CD8 uptake displayed a substantial concentration of densely packed tumor regions. **I-J**) IF staining for CD4 or CD8 in 4T1 tumor sections displayed a notable presence of CD4 + or CD8 + cells in proximity to high cellular tumor regions, a pattern consistent with the uptake pattern of the PET tracer

Representative central slices from PET images and autoradiography showed a similar distribution pattern of activity (Fig. 6A, B, E, F). Tumors with high SUV_mean_ had lower intratumoral heterogeneity (Fig. 6A-D), showing how heterogeneity of signal uptake is independent of SUV. H&E staining (Fig. 6G-H) showed that [^89^Zr]Zr-DFO-CD4 or [^89^Zr]Zr-DFO-CD8 uptake was in the non-necrotic regions of the tumor, indicating there was minimal non-specific radiotracer retention caused due to tumor necrosis. IF staining (Fig. 6I, J) validates the matched positive signal patterns between [^89^Zr]Zr-DFO-CD4 or [^89^Zr]Zr-DFO-CD8 uptake and CD4 or CD8 staining. Taken together, our study suggests that PET imaging can be indicative of the underlying distribution of CD4 or CD8 cells.

## Discussion

Immuno-PET imaging can be a valuable tool for understanding the kinetics and spatial heterogeneity of infiltrating immune cells during immunotherapy by checkpoint blockade. Here we monitored intratumoral and splenic CD8 + or CD4 + T cells longitudinally in response to ICB treatment in immunologically “cold” breast tumors using [^89^Zr]Zr-DFO-CD8 or [^89^Zr]Zr-DFO-CD4 PET imaging. We showed that sustained intratumoral infiltration by CD8 + or CD4 + T cells and decreased spatial heterogeneity within tumors were associated with better ICB treatment outcomes. These findings are consistent with earlier findings based on the analysis of distinct cell populations at necropsy and with the results of clinical trials [4, 7, 10]. While a large CD8 + T cell population at baseline has been shown to correlate with better ICB treatment outcomes in immune-rich environments [26], the responding immunologically “cold” tumors did not show an overall increase in the CD8 signal at baseline. However, an evaluation of the spatial heterogeneity of CD4 + and CD8 + T cells showed that responders had a less heterogeneous distribution of intratumoral CD8 + and CD4 + T cells compared to non-responders. Further, this study shows that increased CD4 and decreased CD8 signaling in the spleen may be predictive biomarkers of response. Taken together, these results show differences in the kinetics of CD4 + and CD8 + T cell proliferation and trafficking in response to ICB treatment and suggest distinct underlying mechanisms to explain the impact of α-PD1 and α-CTLA4 treatment on immunologically “cold” breast tumors.

CD8-PET imaging in immunologically “hot” tumors has shown that baseline intratumoral CD8 + T cell populations are positively correlated with the efficacy of ICB treatment [27]; however, this does not apply to immunologically “cold” tumors. This may be due to differences in the tumor microenvironments (TME) as “cold” tumors have fewer infiltrating immune cells in a predominately suppressive TME, with mostly ‘exhausted’ CD8 + lymphocytes [28]. Furthermore, Dammeijer et al. reported that targeting CD8 + T cells only in the draining lymph nodes but not within the tumor (where CD8 + T cells are exhausted) with α-PD-1 treatment improved CD8 + T cell tumor infiltration and suppressed tumor size [29]. Our study supports the principle that in immunologically “cold” tumors, existing intratumoral CD8 + T cells may not be the main targets of ICB treatment. Therefore, quantification of TIL numbers prior to initiation of treatment may not help in predicting ICB responders. Instead, we observed increased retention of the intratumoral CD8 PET signal in conjunction with a reduced signal in the spleen. While the egress of CD8 + T cells from the spleen and influx into the tumor is a potential mechanism of action to explain the effectiveness of ICB in “cold” tumors, activation of extra-tumoral CD8 + T cells and the increase in tumor infiltration is consistent with the model proposed by Dammeijer et al. [29]. A current clinical trial of CD8 PET imaging uses a single time point (∼ 1 week post-initiation of ICB treatment) to assess the nature of the intratumoral CD8-specific PET signal [8]. In our study, the changes from baseline to early time points of treatment indicated a better predictive outcome in “cold” tumors compared to the reliance on a single time point. Overall, CD8-specific PET imaging showed that the total amount and intratumoral distribution of CD8 + cells in the tumor and the spleen (both before and after treatment) may be important factors to explore in future clinical trials.

CD4 populations have recently gained traction in their role in ICB response. CD4-specific PET showed a significantly increased intratumoral signal shortly after treatment started (day 2). PET imaging for CD4 T cells soon after initiation of ICB might help predict long-term efficacy. A single imaging timepoint might be sufficient to reach that conclusion. Kristensen et al. concluded that CD4-PET imaging could predict the response to α-PD1 treatment by combining data from multiple tumor models, irrespective of tumor types [27]. Nagasaki et al. reported that infiltration by CD4+, but not CD8 + T cells, was a good prognostic factor in classic Hodgkin lymphoma [30]. In our study, the ICB responders had significantly elevated CD4 SUV_peak_ before treatment in the 4T1 model, indicating that the presence of CD4 + intratumoral cells prior to treatment might be a positive prognostic indicator for ICB treatment. Traditionally, immune checkpoint inhibitors were believed to primarily target CD8 + cytotoxic T cells [31]; yet, a recent study demonstrated that α-PD1 can reactivate exhausted intratumoral CD4 + cytotoxic T cells in humans [10]. In our study, it was shown that α-PD1 increases intratumoral CD4 + signal, supporting the mechanisms describing intratumoral CD4 + cells as one of the main targets of α-PD1 treatment. Further, the splenic CD4 signal in the responder group was elevated, suggesting an expansion of the CD4 + cell population. Zuazo et al., showed that circulating CD4 + T cells were a biomarker and key contributor to the success of PD1/PD-L1 blockade [11, 32]. Furthermore, α-CTLA4 impacts the tumor microenvironment in at least two different ways: (1) by depleting regulatory T-cells via antibody-dependent cellular cytotoxicity (ADCC) [33] or by disturbed glycolysis [34]; (2) by activating T helper populations [9, 35]. Nevertheless, Sharma et al. reported that α-CTLA-4 does not deplete regulatory T-cells in human cancers [36]. We found that the splenic CD4 population increased during α-CTLA4 treatment, indicating that in “cold” tumors the activation of T helper cells might play an important role.

Spatial and temporal heterogeneity of immune cell profiles have been associated with the efficacy of immunotherapy in both preclinical and clinical studies in multiple cancer types [37–40]. Most of these studies relied on invasive biopsies or *ex-vivo* analysis, which precluded quantification of heterogeneity across the entire tumor or longitudinal observation. The ability to track spatial of CD4 and CD8 heterogeneity in conjunction with an assessment of the final outcome in “cold” tumors is critical, as the response rate is quite low. In “cold” tumors, increased spatial heterogeneity of TILs, as assessed by IHC, is correlated with unfavorable outcomes [41]. CD4 + or CD8 + T cell clusters can give rise to tertiary lymphoid structures (TLS), which play a pivotal role in tumor-specific immune responses [42]. Although TLSs are prognostic indicators of a favorable response across most cancer types, this is not always the case, as in breast cancer [42]. TLS-associated Tregs and TLS-resident B cells, which can suppress tumor-specific immune responses, may be to blame. To quantify the spatial heterogeneity of CD8 + T cells in 3D, our approach was adopted from Rashidian et al., who analyzed CD8 + cell distribution by PET imaging with a central line method [18]. Our 3D approach showed that low heterogeneity of the distribution of CD8 + T cells, both pre- and post-initiation of ICB treatment, was associated with better therapeutic outcomes. Important to note, a high degree of heterogeneity of the intratumoral CD8 PET signal did not necessarily correspond to a high SUV_mean_. The analysis of the spatial distribution of the PET signal may add critical information not available from a standard assessment of SUV alone. Our findings are consistent with previous invasive (biopsy) studies, which showed that tumors with large numbers of tumor-infiltrating lymphocytes (TILs) may show either a high or low degree of heterogeneity [43]. Heterogeneity in the tumor microenvironment is one of the critical hallmarks of cancer [1]. It impacts tumor responses for a range of therapies including targeted therapy and chemotherapy [43–45]. Therefore, studying the heterogeneity across the entire tumor by immunoPET imaging may improve our understanding of the response to drugs and/or resistance to them, and thus provide us with new ideas for unique combinational therapies.

Despite these findings, questions remain. First, the observed changes in PET signals during ICB treatment may not be attributed solely to TILs since other cell types are known to express CD4 or CD8 (such as CD4 + macrophages, CD8 + dendritic cells, and CD8 + NK cells). This challenge can be partially mitigated through the utilization of RAG-deficient mice, which lack both T and B cells. However, given that these T and B cell deficient mice are unlikely to exhibit a response to ICB treatment, our ability to discern how these remaining signals would manifest in response to ICB is constrained. The simultaneous expression of numerous extracellular receptors and intracellular transcription factors differentiates these distinctive subgroups of immune cells. This multifaceted trait can be identified through invasive methods such as histological validation and flow cytometry. However, those results are unable to be paired with long-term responses. Second, while there were consistencies across models, there were some trends that differed between the MMTV-HER2 and 4T1 models. These two mouse models have different genetic backgrounds, FVB for MMTV-HER2 and Balb/c for 4T1. The CD4 T cell compartment of the FVB strain is known to be further Th2 skewed than that of Balb/c mice [46, 47]. While the impact of the Th1 or Th2 environment on immune profiles and ICB response remains unclear, it is established that Th1 cells eradicate the tumor mass through the induction of cellular immunity, while Th2 cells destroy tumors by inducing tumor necrosis [48]. Further, there are differences in the tumor cells as well: MMTV-HER2 is a gene knock-in model with human HER2 expression [19, 49], while 4T1 is a spontaneous mouse mammary fat pad tumor. HER2 is known to promote cancer cell proliferation and survival as well as secretion of CCL2, a chemokine that recruits monocytes and macrophages and inhibits M1-like macrophage polarization [50–54]. How HER2 immunogenicity and its intrinsic signaling properties affect the response to ICB treatment would be an interesting avenue for future exploration. There are currently clinical trials (NCT04789096, NCT03125928) examining ICB in combination with anti-HER2 therapies. While there are responses, they are typically less than those published for TNBC [12, 55]. Finally, in certain ICB-treated groups, only a small fraction of responders (∼ 10%) could be identified, which posed limitations to statistical analysis. Nevertheless, such a response rate is consistent with what is observed in clinical studies of breast cancer [56].

## Conclusions

In summary, this study provides evidence that increased tumor infiltration by CD4 + and CD8 + T cells and reduced spatial heterogeneity of these T cell populations are key markers for a response to ICB. PET imaging metrics may provide early predictive biomarkers to evaluate the kinetics of TILs in “cold” tumors. We show that, before initiation of treatment of “cold” tumors, intratumoral heterogeneity of the distribution of CD4 + or CD8 + T cells at baseline, not the total number of T cells, is predictive of immunotherapy outcomes in these mouse breast cancer models. Further, sustained infiltration by CD8 + or CD4 + T cells, along with reduced intratumoral heterogeneity, is correlated with enhanced efficacy of immunotherapy. ImmunoPET imaging allows systemic tracking of immune cells in the intact organism during treatment. We show a correlation between a reduction in the splenic CD8-PET signal and an elevation in the splenic CD4-PET signal and an improved response to immunotherapy. PET imaging provides quantitative metrics that correlate with differences in the anti-tumor responses, and also uncovers variations in the mechanism of action of ICB drugs in different host and tumor environments.

### Electronic supplementary material

Below is the link to the electronic supplementary material.

Supplementary Material 1

## Data Availability

The original DICOM files for PET/CT scanning that support the findings of this study are available from the corresponding author, [A.G.S], upon request.

## Abbreviations

CT: Computed tomography
PET: Positron emission tomography
DFO: Deferoxamine
TNBC: Triple-negative breast cancer
ICB: Immune checkpoint blockade
TILs: Tumor-infiltrating lymphocytes
CTLA4: Cytotoxic T lymphocyte-associated antigen 4
PD-1: Programmed cell death 1
ROI: Region of interest
SUV_peak_: Peak of standardized uptake value
SUV_mean_: Mean of standardized uptake value
TME: Tumor microenvironment
IHC: Immunohistochemistry
H&E: Hematoxylin and eosin
IF: Immunofluorescence

## References

[1] Hanahan D (2022). Hallmarks of Cancer: New dimensions. Cancer Discov.

[2] Semiglazov V, Tseluiko A, Kudaybergenova A, Artemyeva A, Krivorotko P, Donskih R (2022). Immunology and immunotherapy in breast cancer. Cancer Biol Med.

[3] Nathan MR, Schmid P (2018). The emerging world of breast cancer immunotherapy. Breast (Edinburgh Scotland).

[4] Farhood B, Najafi M, Mortezaee K (2019). CD8(+) cytotoxic T lymphocytes in cancer immunotherapy: a review. J Cell Physiol.

[5] Borst J, Ahrends T, Babala N, Melief CJM, Kastenmuller W (2018). CD4(+) T cell help in cancer immunology and immunotherapy. Nat Rev Immunol.

[6] Larimer BM, Wehrenberg-Klee E, Dubois F, Mehta A, Kalomeris T, Flaherty K (2017). Granzyme B PET imaging as a predictive biomarker of Immunotherapy Response. Cancer Res.

[7] Paijens ST, Vledder A, de Bruyn M, Nijman HW (2021). Tumor-infiltrating lymphocytes in the immunotherapy era. Cell Mol Immunol.

[8] Farwell MD, Gamache RF, Babazada H, Hellmann MD, Harding JJ, Korn R (2022). CD8-Targeted PET imaging of Tumor-infiltrating T cells in patients with Cancer: a phase I first-in-humans study of (89)Zr-Df-IAB22M2C, a radiolabeled Anti-CD8 Minibody. J Nucl Med.

[9] Tay RE, Richardson EK, Toh HC (2021). Revisiting the role of CD4(+) T cells in cancer immunotherapy-new insights into old paradigms. Cancer Gene Ther.

[10] Balança CC, Salvioni A, Scarlata CM, Michelas M, Martinez-Gomez C, Gomez-Roca C et al. PD-1 blockade restores helper activity of tumor-infiltrating, exhausted PD-1hiCD39 + CD4 T cells. JCI Insight 2021;6. 10.1172/jci.insight.142513 PMC7934837 33332284

[11] Zuazo M, Arasanz H, Bocanegra A, Fernandez G, Chocarro L, Vera R (2020). Systemic CD4 immunity as a key contributor to PD-L1/PD-1 Blockade Immunotherapy Efficacy. Front Immunol.

[12] Marra A, Viale G, Curigliano G (2019). Recent advances in triple negative breast cancer: the immunotherapy era. BMC Med.

[13] Tavaré R, Escuin-Ordinas H, Mok S, McCracken MN, Zettlitz KA, Salazar FB (2016). An effective Immuno-PET Imaging Method to monitor CD8-Dependent responses to Immunotherapy. Cancer Res.

[14] Pesapane F, Suter MB, Rotili A, Penco S, Nigro O, Cremonesi M (2020). Will traditional biopsy be substituted by radiomics and liquid biopsy for breast cancer diagnosis and characterisation?. Med Oncol.

[15] Miladinova D (2019). Molecular imaging in breast Cancer. Nucl Med Mol Imaging.

[16] Ma G, Liu C, Lian W, Zhang Y, Yuan H, Zhang Y (2021). 18)F-FLT PET/CT imaging for early monitoring response to CDK4/6 inhibitor therapy in triple negative breast cancer. Ann Nucl Med.

[17] Lu Y, Li M, Massicano AVF, Song PN, Mansur A, Heinzman KA et al. [(89)Zr]-Pertuzumab PET Imaging reveals Paclitaxel Treatment Efficacy is positively correlated with HER2 expression in human breast Cancer Xenograft Mouse models. Molecules 2021;26. 10.3390/molecules26061568 PMC8001650 33809310

[18] Rashidian M, Ingram JR, Dougan M, Dongre A, Whang KA, LeGall C (2017). Predicting the response to CTLA-4 blockade by longitudinal noninvasive monitoring of CD8 T cells. J Exp Med.

[19] Hanker AB, Estrada MV, Bianchini G, Moore PD, Zhao J, Cheng F (2017). Extracellular Matrix/Integrin Signaling Promotes Resistance to combined inhibition of HER2 and PI3K in HER2(+) breast Cancer. Cancer Res.

[20] Gallegos C, lu Y, Clements J, Song P, Lynch S, Mascioni A (2024). [ 89 Zr]-CD8 ImmunoPET imaging of glioblastoma multiforme response to combination oncolytic viral and checkpoint inhibitor immunotherapy reveals CD8 infiltration differential changes in preclinical models. Theranostics.

[21] Lu Y, Massicano AVF, Gallegos CA, Heinzman KA, Parish SW, Warram JM, et al. Evaluating the Accuracy of FUCCI Cell cycle in vivo fluorescent imaging to assess Tumor Proliferation in Preclinical Oncology models. Mol Imaging Biol; 2022. 10.1007/s11307-022-01739-9 35650411

[22] Snipstad S, Bremnes F, Dehli Haugum M, Sulheim E (2023). Characterization of immune cell populations in syngeneic murine tumor models. Cancer Med.

[23] Perrone M, Talarico G, Chiodoni C, Sangaletti S. Impact of Immune Cell Heterogeneity on HER2 + breast Cancer prognosis and response to Therapy. Cancers (Basel) 2021;13. 10.3390/cancers13246352 PMC8699132 34944971

[24] Swoboda A, Nanda R (2018). Immune checkpoint blockade for breast Cancer. Cancer Treat Res.

[25] Sher A, Lacoeuille F, Fosse P, Vervueren L, Cahouet-Vannier A, Dabli D (2016). For avid glucose tumors, the SUV peak is the most reliable parameter for [(18)F]FDG-PET/CT quantification, regardless of acquisition time. EJNMMI Res.

[26] Li F, Li C, Cai X, Xie Z, Zhou L, Cheng B (2021). The association between CD8 + tumor-infiltrating lymphocytes and the clinical outcome of cancer immunotherapy: a systematic review and meta-analysis. EClinicalMedicine.

[27] Kristensen LK, Fröhlich C, Christensen C, Melander MC, Poulsen TT, Galler GR (2019). CD4(+) and CD8a(+) PET imaging predicts response to novel PD-1 checkpoint inhibitor: studies of Sym021 in syngeneic mouse cancer models. Theranostics.

[28] Liu YT, Sun ZJ (2021). Turning cold tumors into hot tumors by improving T-cell infiltration. Theranostics.

[29] Dammeijer F, van Gulijk M, Mulder EE, Lukkes M, Klaase L, van den Bosch T (2020). The PD-1/PD-L1-Checkpoint restrains T cell immunity in Tumor-Draining Lymph Nodes. Cancer Cell.

[30] Nagasaki J, Togashi Y, Sugawara T, Itami M, Yamauchi N, Yuda J (2020). The critical role of CD4 + T cells in PD-1 blockade against MHC-II-expressing tumors such as classic Hodgkin lymphoma. Blood Adv.

[31] Wei SC, Duffy CR, Allison JP (2018). Fundamental mechanisms of Immune Checkpoint Blockade Therapy. Cancer Discov.

[32] Zuazo M, Arasanz H, Bocanegra A, Chocarro L, Vera R, Escors D (2020). Systemic CD4 immunity: a powerful clinical biomarker for PD-L1/PD-1 immunotherapy. EMBO Mol Med.

[33] Simpson TR, Li F, Montalvo-Ortiz W, Sepulveda MA, Bergerhoff K, Arce F (2013). Fc-dependent depletion of tumor-infiltrating regulatory T cells co-defines the efficacy of anti-CTLA-4 therapy against melanoma. J Exp Med.

[34] Zappasodi R, Serganova I, Cohen IJ, Maeda M, Shindo M, Senbabaoglu Y (2021). CTLA-4 blockade drives loss of T(reg) stability in glycolysis-low tumours. Nature.

[35] Lichtman AH. A role for T helper cells in anti-CTLA-4 therapy. Sci Immunol 2017;2. 10.1126/sciimmunol.aao6871 28864497

[36] Sharma A, Subudhi SK, Blando J, Scutti J, Vence L, Wargo J (2019). Anti-CTLA-4 Immunotherapy does not deplete FOXP3(+) Regulatory T cells (Tregs) in human cancers. Clin Cancer Res.

[37] Jia Q, Wang A, Yuan Y, Zhu B, Long H (2022). Heterogeneity of the tumor immune microenvironment and its clinical relevance. Exp Hematol Oncol.

[38] Lin Z, Meng X, Wen J, Corral JM, Andreev D, Kachler K (2020). Intratumor Heterogeneity correlates with reduced Immune Activity and worse survival in Melanoma patients. Front Oncol.

[39] Li J, Byrne KT, Yan F, Yamazoe T, Chen Z, Baslan T et al. Tumor Cell-intrinsic factors underlie heterogeneity of Immune Cell infiltration and response to Immunotherapy. Immunity 2018;49:178 – 93.e7. 10.1016/j.immuni.2018.06.006 PMC6707727 29958801

[40] Minnema-Luiting J, Vroman H, Aerts J, Cornelissen R. Heterogeneity in Immune Cell Content in Malignant Pleural Mesothelioma. Int J Mol Sci 2018;19. 10.3390/ijms19041041 PMC5979422 29601534

[41] Jung M, Lee JA, Yoo SY, Bae JM, Kang GH, Kim JH (2022). Intratumoral spatial heterogeneity of tumor-infiltrating lymphocytes is a significant factor for precisely stratifying prognostic immune subgroups of microsatellite instability-high colorectal carcinomas. Mod Pathol.

[42] Schumacher TN, Thommen DS (2022). Tertiary lymphoid structures in cancer. Science.

[43] Marusyk A, Janiszewska M, Polyak K (2020). Intratumor Heterogeneity: the Rosetta Stone of Therapy Resistance. Cancer Cell.

[44] Zhang A, Miao K, Sun H, Deng CX (2022). Tumor heterogeneity reshapes the tumor microenvironment to influence drug resistance. Int J Biol Sci.

[45] Janku F (2014). Tumor heterogeneity in the clinic: is it a real problem?. Ther Adv Med Oncol.

[46] Zhang BB, Yan C, Fang F, Du Y, Ma R, Li XY (2017). Increased hepatic Th2 and Treg subsets are associated with biliary fibrosis in different strains of mice caused by Clonorchis sinensis. PLoS ONE.

[47] Kim EM, Bae YM, Choi MH, Hong ST (2012). Cyst formation, increased anti-inflammatory cytokines and expression of chemokines support for Clonorchis sinensis infection in FVB mice. Parasitol Int.

[48] Nishimura T, Iwakabe K, Sekimoto M, Ohmi Y, Yahata T, Nakui M (1999). Distinct role of antigen-specific T helper type 1 (Th1) and Th2 cells in tumor eradication in vivo. J Exp Med.

[49] Hanker AB, Pfefferle AD, Balko JM, Kuba MG, Young CD, Sánchez V (2013). Mutant PIK3CA accelerates HER2-driven transgenic mammary tumors and induces resistance to combinations of anti-HER2 therapies. Proc Natl Acad Sci U S A.

[50] Yang H, Zhang Q, Xu M, Wang L, Chen X, Feng Y (2020). CCL2-CCR2 axis recruits tumor associated macrophages to induce immune evasion through PD-1 signaling in esophageal carcinogenesis. Mol Cancer.

[51] Gschwandtner M, Derler R, Midwood KS (2019). More than just attractive: how CCL2 influences myeloid cell Behavior Beyond Chemotaxis. Front Immunol.

[52] Triulzi T, Forte L, Regondi V, Di Modica M, Ghirelli C, Carcangiu ML (2019). HER2 signaling regulates the tumor immune microenvironment and trastuzumab efficacy. Oncoimmunology.

[53] Jin J, Lin J, Xu A, Lou J, Qian C, Li X (2021). CCL2: an important mediator between Tumor cells and Host Cells in Tumor Microenvironment. Front Oncol.

[54] Rogic A, Pant I, Grumolato L, Fernandez-Rodriguez R, Edwards A, Das S (2021). High endogenous CCL2 expression promotes the aggressive phenotype of human inflammatory breast cancer. Nat Commun.

[55] Agostinetto E, Montemurro F, Puglisi F, Criscitiello C, Bianchini G, Del Mastro L et al. Immunotherapy for HER2-Positive breast Cancer: clinical evidence and future perspectives. Cancers (Basel) 2022;14. 10.3390/cancers14092136 PMC9105460 35565264

[56] Keenan TE, Tolaney SM (2020). Role of Immunotherapy in Triple-negative breast Cancer. J Natl Compr Canc Netw.

