# Supplementary material for: Evaluating the immunologically “cold” tumor microenvironment after treatment with immune checkpoint inhibitors utilizing PET imaging of CD4 + and CD8 + T cells in breast cancer mouse models
Source: Breast Cancer Res. 2024 Jun 25;26:104. doi: 10.1186/s13058-024-01844-3 (PMC11201779; doi:10.1186/s13058-024-01844-3)
Supplement: Supplementary file 1 — Supplementary Material 1 [file 13058_2024_1844_MOESM1_ESM.docx]

**Supplemental data for**

**“Evaluating “cold” tumor immune microenvironment after immune checkpoint blockage treatment via [^89^Zr]Zr-DFO-CD4 and [^89^Zr]Zr-DFO-CD8 PET imaging in breast cancer mouse models”**

**Supplemental Results:**

***Blocking experiments validated the specificity of [^89^Zr]Zr-DFO-CD4 and [^89^Zr]Zr-DFO-CD8 PET imaging***

A blocking experiment was conducted to validate the specificity of the CD4 and CD8 radiotracers (Supplemental Results and Supplemental Figure 1). The results showed that in naïve immunocompetent mice, [^89^Zr]Zr-DFO-CD4 and [^89^Zr]Zr-DFO-CD8 were mainly uptake by lymph nodes (**Supplemental Figure 1A**, red arrows), and spleens (**Supplemental Figure 1A**, yellow arrows). With blocking agents, the radiotracer uptake in lymph nodes and spleens was largely decreased (**Supplemental Figure 1A**). The mean of standard uptake value (SUV_mean_) in the spleen showed a significant 3-fold decrease after blocking (**Supplemental Figure 1B-C**, p<0.001 for [^89^Zr]Zr-DFO-CD4 and p<0.01 for [^89^Zr]Zr-DFO-CD8). Biodistribution showed there was a significant decrease of [^89^Zr]Zr-DFO-CD4 uptake with blocking in the spleen (p<0.05), small intestine (p<0.05), and large intestine (**Supplemental Figure 1D**, p<0.01). In [^89^Zr]Zr-DFO-CD8 biodistribution, spleen uptake was decreased with blocking, along with increased uptake in blood and heart (p<0.01), indicating the radiotracers remained in the circulation when not uptake by tissues (**Supplemental Figure 1E**). These results indicated [^89^Zr]Zr-DFO-CD4 and [^89^Zr]Zr-DFO-CD8 were specifically targeting immunological organs and tissues.


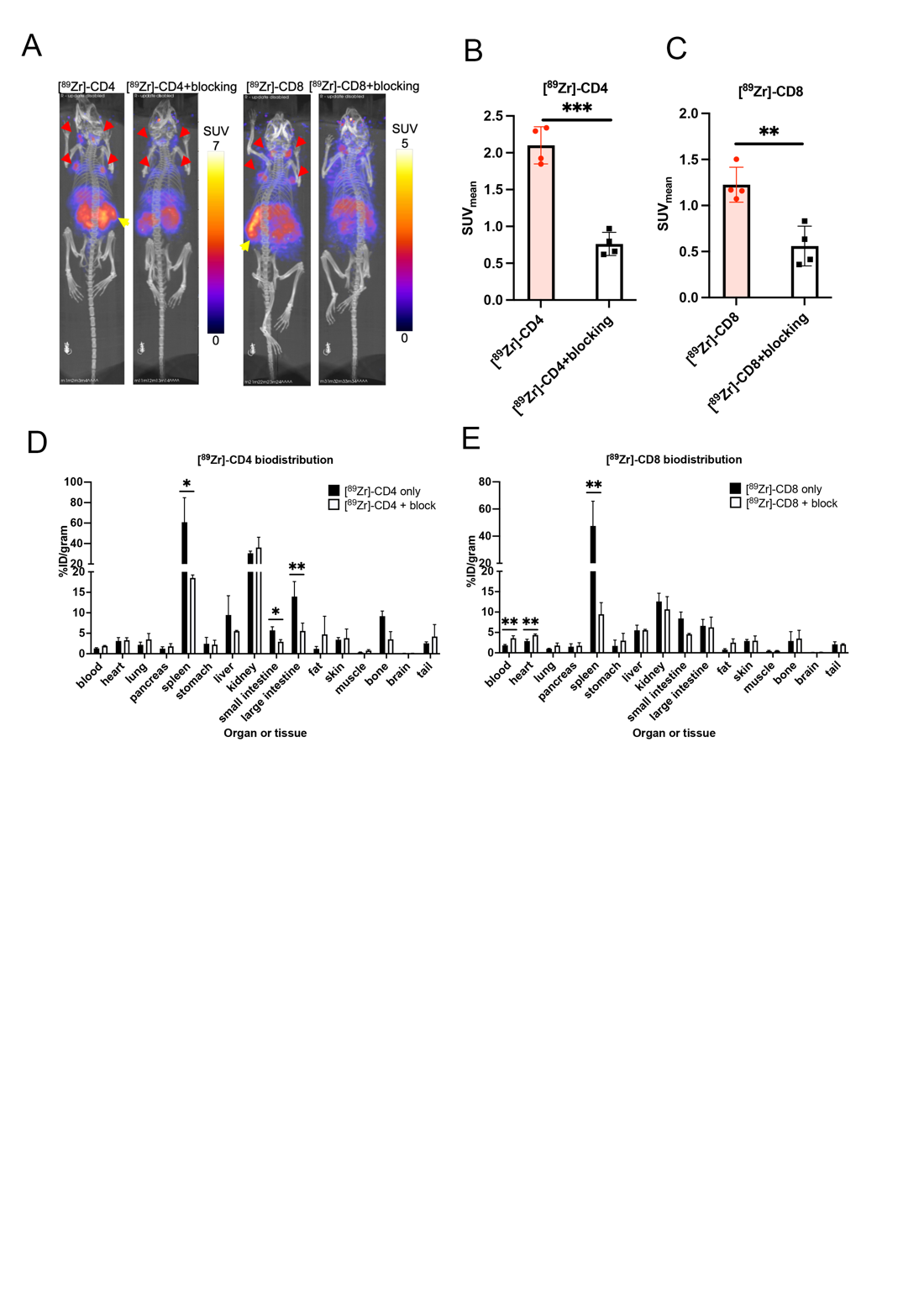


**Supplemental Figure 1.** **Blocking and biodistribution experiments confirmed the specificity of [^89^Zr]Zr-DFO-CD4 and [^89^Zr]Zr-DFO-CD8 minibody radiotracer.** **A**) Representative images of [^89^Zr]Zr-DFO-CD4 and [^89^Zr]Zr-DFO-CD8 PET imaging. The blocking groups showed a great reduction of radiotracer uptake in lymph nodes and spleens. Red arrows: lymph nodes; yellow arrows: spleens. **B-C**) The mean of standard uptake value (SUV) in the spleen. blocking group showed significantly reduced [^89^Zr]Zr-DFO-CD4 SUV_mean_ (**B**) and [^89^Zr]Zr-DFO-CD8 SUV_mean_ (**C**). **D-E**) Biodistribution of [^89^Zr]Zr-DFO-CD4 (**D**) and [^89^Zr]Zr-DFO-CD8 (**E**) in mouse organs and tissues. Blocking groups showed significantly reduced radiotracer uptake. N=4 for each group. ns, non-significant; *, p<0.05; **, p<0.01.


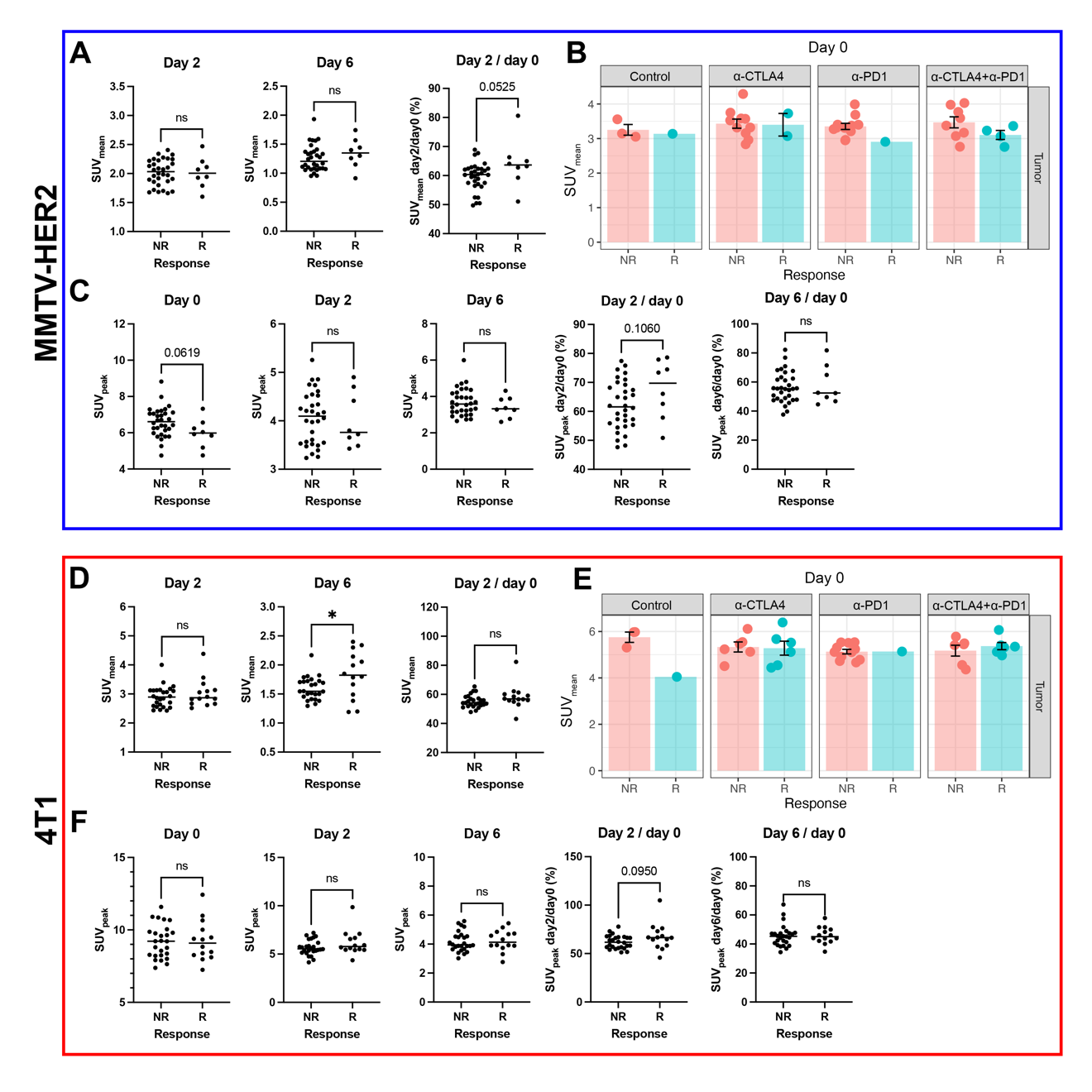


**Supplemental Figure 2. The dynamic changes of intratumoral CD8+ cells measured by [^89^Zr]Zr-DFO-CD8 during ICB treatment. A**) Mean of standard uptake value (SUV_mean_) on day 2, 6, and the ratio of day 2/day 0 in MMTV-HER2 model. **B**) Baseline intratumoral [^89^Zr]Zr-DFO-CD8 SUV_mean_ of each treatment group in MMTV-HER2 model. No significant differences were observed. **C**) 3x3x3 regional peak of standard uptake value (SUV_peak_) at day 0, 2, 6 in MMTV-HER2 model. No significant differences were observed between non-responders and partial responders of SUV_peak_. **D**) Mean of standard uptake value (SUV_mean_) on days 2, 6, and ratio of day 2/day 0 in the 4T1 model. **E**) Baseline intratumoral [^89^Zr]Zr-DFO-CD8 SUV_mean_ of each treatment group in the 4T1 model. **F**) [^89^Zr]Zr-DFO-CD8 SUV_peak_ from day 0 to 6 in 4T1 models. No significant changes were observed. N=80. ns, non-significant; *, P<0.05.


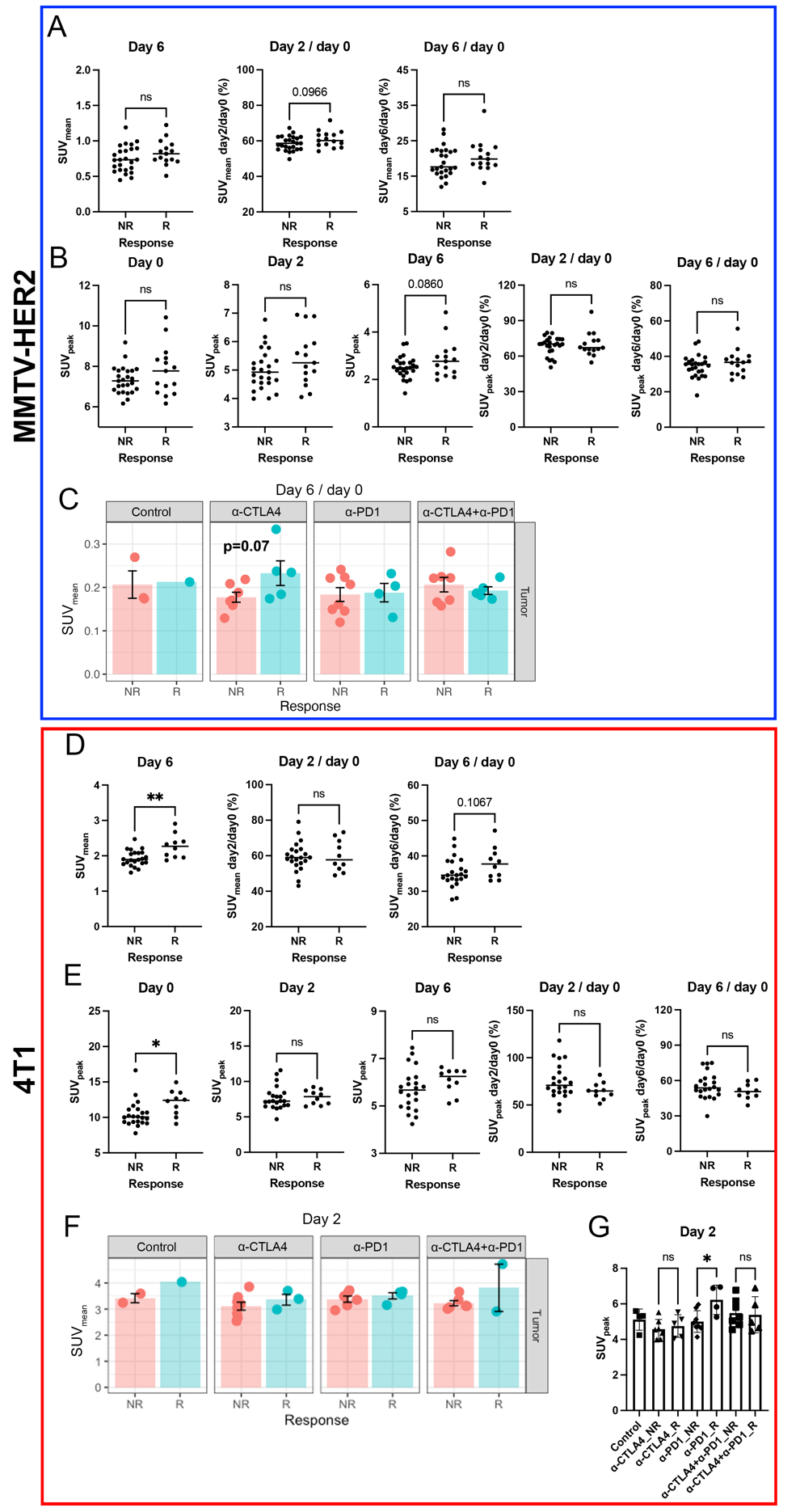


**Supplemental Figure 3. The dynamic changes of intratumoral CD4+ cells measured by [^89^Zr]Zr-DFO-CD4. A**) SUV_mean_ on day 0, 6, and ratio of day2/day0 and day6/day0 in MMTV-HER2 model. **B**) [^89^Zr]Zr-DFO-CD4 SUV_peak_ from day 0 to 6 in MMTV-HER2 models. No significant changes were observed. **C**) Day6/day0 intratumoral [^89^Zr]Zr-DFO-CD4 SUV_mean_ of each treatment group in MMTV-HER2 model. **D**) [^89^Zr]Zr-DFO-CD4 SUV_mean_ at day 0, 6, and the ratio of day2/day0 and day6/day0 in the 4T1 model. **E**) [^89^Zr]Zr-DFO-CD4 SUV_peak_ from day 0 to 6 in 4T1 models. No significant changes were observed. **F**) Day 2 intratumoral [^89^Zr]Zr-DFO-CD4 SUV_mean_ of each treatment group in the 4T1 model. No significant differences were observed. **G**) [^89^Zr]Zr-DFO-CD4 SUV_peak_ showed a significant increase on day 2 with α-PD1 treatment in the MMTV-HER2 model. N=80. ns, non-significant; *, p<0.05.


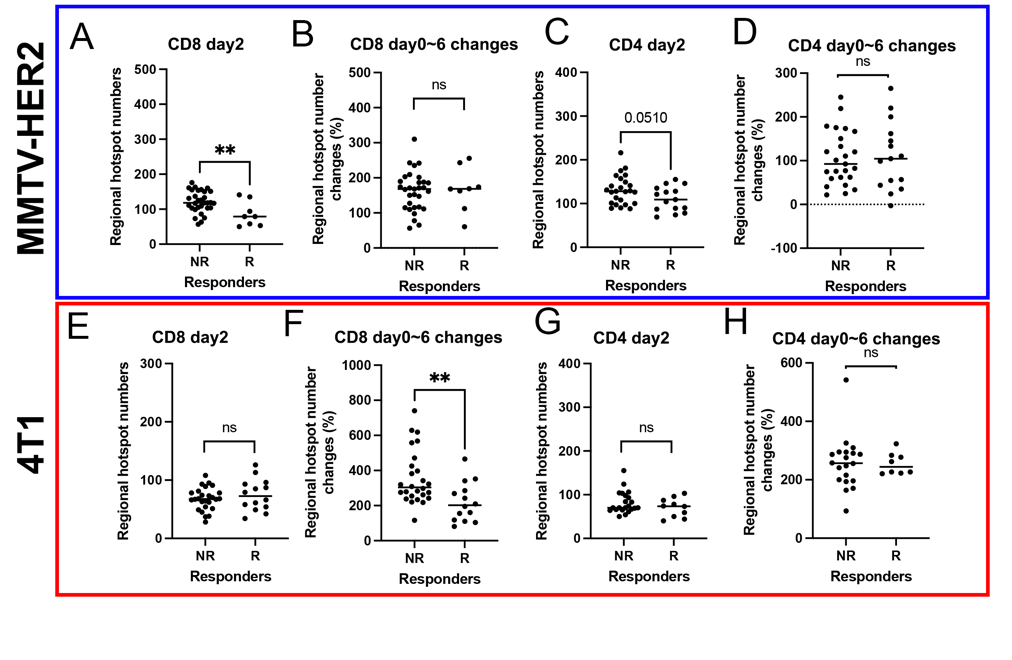


**Supplemental Figure 4. Heterogeneity analysis showed Intratumoral heterogeneity of CD4 or CD8 signal.** **A**) CD8 or CD4 heterogeneity analysis of MMTV-HER2 tumors. On day 2, the responders showed decreased regional hotspot numbers of [^89^Zr]Zr-DFO-CD8 uptake compared to non-responders. **B**) CD8 or CD4 heterogeneity analysis of 4T1 tumors. From day 0 to day 6, the responders showed decreased regional hotspot numbers of [^89^Zr]Zr-DFO-CD8 uptake compared to non-responders.


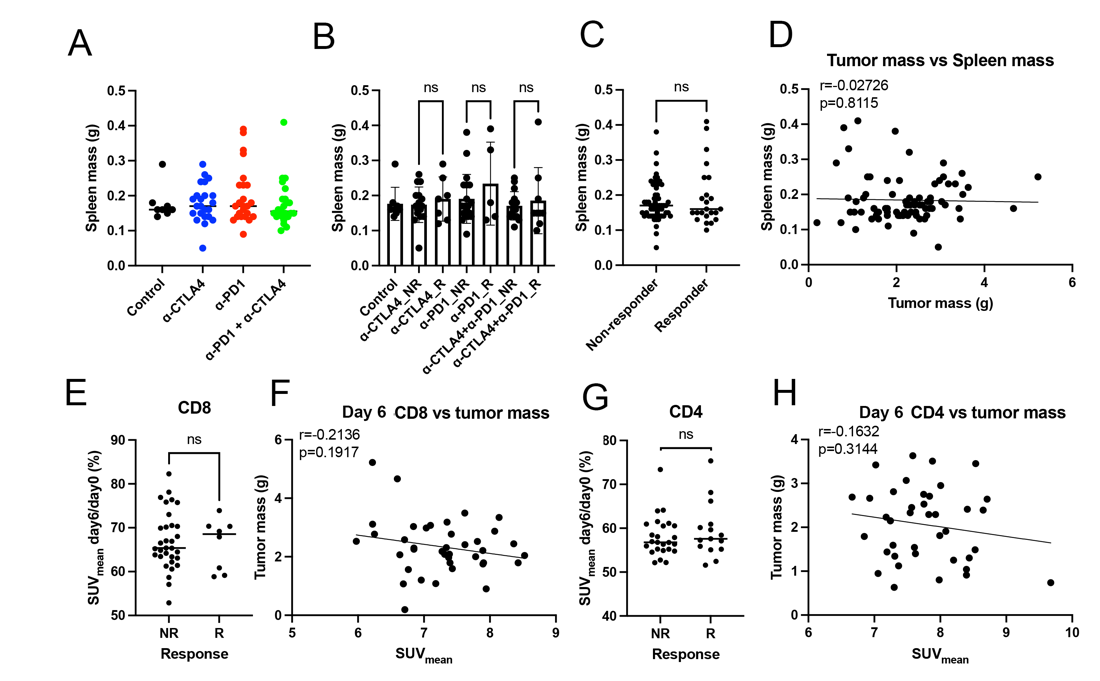


**Supplemental Figure 5. The splenic CD8+ or CD4+ signal did not correlate with terminal spleen mass in the MMTV-HER2 model.** **A-C**) Terminal spleen mass in different ICB treatment groups and responses in MMTV-HER2 model. No significant differences were observed. **D**) In the MMTV-HER2 model, there was no significant correlation between terminal tumor mass and spleen mass. **E-H**) In the MMTV-HER2 model, there was no significant difference between ICB responders and non-responders in splenic [^89^Zr]Zr-DFO-CD8 (**E**) and [^89^Zr]Zr-DFO-CD4 (**G**) SUV_mean_ changes from day 0 to day 6. In the MMTV-HER2 model, there was no significant correlation between [^89^Zr]Zr-DFO-CD8 SUV_mean_ changes and terminal tumor mass (**F**) or day 6 [^89^Zr]Zr-DFO-CD4 SUV_mean_ and terminal tumor mass (**H**).
